# Application of Technology in Cardiopulmonary Resuscitation, a Narrative Review

**DOI:** 10.3390/jcm12237383

**Published:** 2023-11-29

**Authors:** Catherine V. Levitt, Kirsten Boone, Quincy K. Tran, Ali Pourmand

**Affiliations:** 1 Department of Emergency Medicine, George Washington University School of Medicine and Health Sciences, Washington, DC 20037, USA; catherinelevitt@gwu.edu (C.V.L.); kboone@mfa.gwu.edu (K.B.); pourmand@gwu.edu (A.P.); 2 Department of Emergency Medicine, University of Maryland School of Medicine, Baltimore, MD 21201, USA; 3 Program in Trauma, The R Adams Cowley Shock Trauma Center, University of Maryland School of Medicine, Baltimore, MD 21201, USA

**Keywords:** cardiopulmonary resuscitation, technology, cardiac arrest

## Abstract

Novel medical technologies are designed to aid in cardiopulmonary resuscitation both in and out of the hospital. Out-of-hospital innovations utilize the skills of paramedics, bystanders, and other prehospital personnel, while in-hospital innovations traditionally aid in physician intervention. Our review of current literature aims to describe the benefits and limitations of six main technologic advancements with wide adoption for their practicality and functionality. The six key technologies include: extracorporeal membrane oxygenation (ECMO), real-time feedback devices, smart devices, video review, point-of-care ultrasound, and unmanned aerial vehicle (drone) automated external defibrillator (AED) delivery. The benefits and limitations of each technology were independently reviewed and expounded upon. Newer technologies like drone AED delivery, paramedic ultrasound use, and smart devices have been demonstrated to be safe and feasible, however, further studies are needed to compellingly demonstrate improved patient outcomes. In-hospital use of ECMO and ultrasound is well established by current literature to aid in cardiopulmonary resuscitation and improve patient outcomes.

## 1. Introduction

Technology is becoming increasingly present both in our everyday lives and in healthcare with numerous applications to the field of cardiac arrest and cardiopulmonary resuscitation (CPR). Out-of-hospital cardiac arrests (OHCAs), which occur predominantly at home (73%) or in a public setting (16%), are 2 times more likely to be fatal than in-hospital cardiac arrests (IHCAs) with a 9% survival rate versus 18% [1]. The high fatality rate is thought to be in part due to the longer time from cardiac arrest to initiation of CPR and to receiving a defibrillated shock.

With the introduction and widespread availability of smartphones, wearable devices, and other technological advances such as automated external defibrillators (AEDs), there is an increase in bystander-initiated CPR and defibrillation rates, leading to improved patient outcomes and overall survival due in part to the faster initiation of CPR and shock administration. Smartphones and watches are also capable of alerting emergency medical services of a potential cardiac arrest, and these devices can then guide bystanders to perform CPR for OHCA using applications (apps) and feedback technology [2]. Drones are also being utilized as a delivery system for AEDs which increases access in rural and remote areas [3]. 

New technologies with an emphasis on feedback mechanisms are being developed to further improve quality metrics of CPR that are administered both in and out of the hospital. Real-time feedback devices such as manikins with audio-visual capabilities, AEDs with verbal prompts and on-screen cues, and even simple metronomes or audible “click” devices have become standard in how we teach and practice CPR in simulation. Additionally, newer research is looking into the utilization of video review of CPR to provide feedback to providers on how best to streamline the process, minimize compression interruption, and more effectively deliver high-quality CPR [4]. 

Technology has also expanded into using adjuncts to provide both additional information and additional support while CPR is ongoing. Within the emergency department the use of point-of-care ultrasound (POCUS) to help identify reversible causes of cardiac arrest can lead to faster diagnosis and more tailored resuscitation [5], while the use of early extracorporeal membrane oxygenation (ECMO) has been shown to help decrease the amount of low-flow time and improve survival and neurologic outcomes in patients [6]. 

This review aims to summarize the current data that exist on the technology available for in and out-of-hospital arrests and its utilization during CPR. These technologies were selected by our team for their wide adoption with practicality and functionality.

## 2. Methods

The PubMed database was searched from the beginning to 12 September 2023, using the medical subject headings (MeSH) “Out-of-Hospital Cardiac Arrest”, “Technology”, “Heart Arrest”, and “Death, Sudden, Cardiac” to find articles for inclusion. We included all types of relevant studies including, but not limited to, observational studies, case series, case reports, narrative reviews, or systematic reviews. We limited our search result to include articles involving human, adult patients, in the English language. The result revealed 160 matches. Initially, two reviewers independently screened titles and abstracts and of which 83 were included in this review. Furthermore, additional references were added at the authors’ discretion. Articles were not included if they were not primarily in English or did not have an English translation, were focused on pediatric patients, or were preliminary/unpublished results. Any discrepancies during the selection of eligible articles were resolved by discussion among the investigators. This study is based on previously conducted studies and does not contain data with human participants or animals performed by any of the authors.

## 3. Results

Table 1 lists the strengths and weaknesses of current technology that is designed to help with CPR.

### 3.1. Extracorporeal Membrane Oxygenation

Utilizing extracorporeal membrane oxygenation (ECMO) during cardiac arrest is termed extracorporeal cardiopulmonary resuscitation (ECPR) and is thought to improve cardiac arrest outcomes by reestablishing circulation prior to return of spontaneous circulation (ROSC) [6]. A literature review by Abrams et al. summarized that in patients who received ECPR compared to high-quality standardized CPR, there was a trend towards higher survival rates with more meaningful neurologic and functional outcomes [6]. In the ARREST trial, which compared survival to hospital discharge between patients with OHCA and with early ECMO initiation upon arrival at the emergency department versus standard advanced cardiac life support (ACLS), it was found that the ECPR group had significantly improved survival and the trial was terminated early due to the superiority of the ECMO group [7]. In the meta-analysis performed by Scquizzato et al., ECPR resulted in increased survival and favorable neurological outcomes in adults with an OHCA, especially if their initial rhythm was shockable [8]. Patient-specific factors such as age greater than 65 years, duration of low-flow time greater than 60 min, initial non-shockable rhythm, and known life-limiting comorbidities result in unfavorable outcomes and increased mortality when ECPR is initiated [6,9]. After 10 min of unsuccessful CPR, providers are encouraged to activate ECPR teams as the cannulation procedure can take anywhere from 40–80 min, and the risk of hypoxic brain injury increases [6,9,10]. The most common reason for cardiac arrest seems to be from cardiac disease, either congenital in the case of infants or children or coronary artery disease in the cases of adults [11]. A higher percentage of ECPR patients undergo percutaneous coronary intervention (PCI) and studies suggest this leads to improved outcomes for patients [6,9]. In patients who had cardiac arrest secondary to hypothermia, ECPR followed by rewarming may be associated with higher survival and favorable neurological outcomes compared to traditional CPR if ECPR is continued until ROSC is achieved, however, ECPR should not be used if the core temperature is greater than 30 degrees Celsius or if there is major trauma [12]. Survival and neurological outcomes of patients receiving ECPR were similar between those with targeted temperature management and those without [13]. The most common adverse effects of receiving ECPR are pulmonary hemorrhage, renal failure, and neurologic injury resulting in high mortality [14]. Extreme hyperoxia (PaO_2_ > 300 mmHg) postcardiac arrest was found to be more commonly associated with ECPR patients compared to CPR and independently associated with a 2.5-fold increased risk of mortality [15]. Furthermore, extreme hyperoxia between 6 and 36 h after cardiac arrest was found to have worse neurologic outcomes and is associated with higher mortality [16]. 

### 3.2. Real-Time Feedback Devices

Feedback devices for CPR range in complexity, from a simple metronome to elaborate devices which provide audiovisual feedback, and these devices can further be divided into those associated or not associated with automated external defibrillators (AEDs) [17]. These AEDs with feedback provide real-time visual feedback of depth, rate, and chest recoil along with verbal prompts. Studies suggest that use of this type of device during CPR results in chest compression depth and rate improvement and higher accuracy [18,19]. In one study, the greatest improvement in CPR quality is observed with the use of an AED with real-time feedback even when performed by individuals with a low level of CPR skill in bystander CPR [20]. 

Cardio First Angel is a portable device which is affixed to the chest of a patient and provides an audible “click” when adequate compression depth and full recoil are reached. In one study involving IHCA patients, the use of this device resulted in significant improvements in CPR quality compared to no device, and ROSC was observed more frequently, with a decrease in rib fractures [17]. Hand placement and chest compression depth were also improved when using this device [21]. 

A CPRmeter is a device which gives real-time feedback related to the depth and rate of chest wall movement during compression and detects whether the patient’s chest is able to fully recoil between compressions. Some studies evaluating the CPRmeter showed stability of force applied after 4 min of CPR or greater and optimization of positioning with improvement in compression depth and rate, however, additional studies suggest that compression quality was inferior to standard CPR and there were delays in initiation of CPR [17,22,23]. One study looked at the accuracy of placement of a “CPR Assist” feedback device by medical personnel and found that approximately half of the time the device was in an inappropriate position on the chest, increasing the risk of sternal and rib fractures [24]. 

While feedback devices are useful tools, they need to be properly used in order to maximize their efficacy. Another study focused on perceived workload of team leaders with and without the use of feedback devices and showed that team leaders had a lower workload when a feedback device is used during CPR, suggesting that team leaders do not feel they need to monitor the CPR quality as closely and can focus on other aspects of the resuscitation [25]. 

Perhaps the most commonly used real-time feedback devices are manikins that are capable of measuring chest compression depth and rate, determining if full chest recoil is permitted, and noting ventilation volume and rate. In a 2017 study performed on laypeople with no prior CPR training, it was found that during a basic life support (BLS)/AED course, individuals who are of male sex or who have higher BMI, weight, or height were less likely to achieve complete chest recoil, however, those same individual characteristics resulted in the rescuer being more likely to achieve correct compression depth [26]. Expanding on that study, combining the same BLS/AED course with real-time visual feedback via manikin resulted in a statistically significant improvement for the percentage of compressions with complete chest recoil, hand position, and compression depth compared to no manikin feedback [27]. Interestingly, the two groups who received manikin feedback for 1 min vs. 10 min did not differ significantly in terms of these results, suggesting that the addition of real-time manikin feedback, and not its duration, is sufficient to improve CPR technique [27]. Subsequent studies also suggest that overall CPR performance on all metrics improves when using manikins, and there is a reduction in “hands-off” time during resuscitations [28,29]. These results are seen when using both adult and infant manikins, but greater improvement has been shown in infant CPR when looking at ventilation volume and rate before and after trainings with feedback-capable manikins [28,30]. In a study by Austin et al., the use of a metronome for CPR training on infant manikins resulted in more ideal compression rate compared to visual feedback, however, visual feedback resulted in more ideal depth of compressions, suggesting that a combination of audio and visual feedback may result in better-quality CPR for infants [31]. Voice-assisted manikins provide immediate feedback and prompts of how to improve technique during training sessions. CPR training using real-time device feedback for nurses and nursing students showed that the accuracy of chest compressions, mouth-to-mouth ventilation, and overall performance ability improved when compared pre- and postdevice feedback and this was statistically significant when compared to a control group of students who received CPR training without the device [32,33,34]. Trainees subjectively prefer the feedback given by devices, noting less bias compared to instructor feedback and stating an increase in motivation to learn and improve the quality of their CPR [28,35]. In medical student training of CPR skills, supplementing visual feedback devices during simulated cardiac arrest resuscitations resulted in an increased ratio of full chest recoil, while maintaining adequate chest compression depth and rate [36,37,38]. A “QCPR Classroom” is a real-time visual feedback system with manikins providing feedback simultaneously to a large group of trainees, and according to Tanaka et al., training in this environment led to more adequate depth of CPR compressions and allowance of full chest recoil when coupled with a metronome sound [39]. 

Smart advanced life support (SALS) is a newer version of the standard ALS protocol with the addition of real-time physician-supervised video calls for OHCA [40,41]. In a study comparing patient outcomes for OHCA before and after implementation of physician assistance and coaching via real-time video calls to EMS providers, the addition of real-time feedback resulted in increased prehospital ROSC, survival to discharge, and favorable neurological outcomes [40]. 

### 3.3. Smart Devices

Smartphone applications (apps) can provide detailed instructions for bystanders to perform high-quality CPR prior to EMS arrival and provide feedback regarding the chest compression depth and rate in real time by using the phone’s acceleration sensor data [42,43]. However, the medical correctness of the information on some of these apps is not well regulated. In Singapore, the “myResponder” app enables EMS dispatchers to link OHCA victims to nearby volunteers using the mobile 4G network and embedded geolocation technology of smartphones. Using the same technology, the app “myResponder” can identify the nearest AED, which reduces the time from event to CPR initiation and defibrillation [44]. Similar apps, such as “Staying Alive” in Paris have replicated these results with significant improvement in survival outcomes for patients who received first responder CPR, presumably due to the accelerated initiation of efficient CPR [45]. Studies in the US have also demonstrated similar results with improvement in CPR start time and early defibrillation [46]. Apps like “iResus”, which provides instant access to algorithms and drug doses for resuscitations, have also been shown to significantly improve physicians’ performance of ALS during simulated medical emergencies and increase confidence in making medical decisions [47]. In a systematic review by An et al., and other studies, the use of smartphones during CPR was found to maintain chest compression rate and depth in a statistically significant way compared to non-users but it was noted to be painful/difficult to use and hold a smartphone while administering CPR [48,49].

The usage of a smartwatch can further help CPR guidance using its vibration function to reinforce accurate depth and rate of compressions, and compared to smartphone apps it has been shown to be more accurate in providing this feedback [42,50]. Use of smartwatches has also been found to be beneficial in improving the quality of CPR regarding rate and depth of chest compressions as compared to non-watch wearers in both adult and infant studies [48,49,51,52]. Smartwatch users also note that they felt more confident in administering CPR while wearing their watch due to the feedback functionality [42]. During active CPR, one study placed smartwatches on the wrist of patients and compared pulse check by physicians at the carotid artery versus pulse check at the wrist by the smartwatch and found 100% specificity of the smartwatch at the wrist in detecting a pulse (ROSC) and that the smartwatch was more accurate in finding a pulse compared to manual palpation by physicians at the carotid [53]. 

Smart glasses are a new type of wearable technology with video-streaming capabilities. In one study which compared chest compression quality performed by individuals wearing and not wearing the glasses, there was no significant difference, however, there was improvement in the ability of glasses wearers to complete the basic life support (BLS) protocol, open an airway, check breathing, position AED pads, and deliver a shock [54]. These results were echoed by another pilot study which found that CPR quality was not improved with the usage of smart glasses but did note that out of 96 skills that were assessed using the smart glasses, assistance by the dispatcher was given in 65 steps (72% of interventions) with the majority of the assistance related to AED usage [55]. 

A smart ring worn on the finger of the user is also being developed and studied as it relates to CPR feedback. One study showed that the smart ring improved chest compression depth and lengthens the amount of time that accurate chest compression depth was reached [56]. The introduction of a ring device may be beneficial in infant CPR in which providers use two fingers, or two thumbs, and in the proof of concept study by Lee et al., the smart ring may be accurate in detecting chest compression depth when using the two-thumb method, but further studies are needed [57]. 

### 3.4. Cardiopulmonary Resuscitation Video Review

Video review of in-hospital traumas has been well established in the literature to have numerous clinical and educational benefits. In both adult and pediatric trauma centers, trauma video review (TVR)-based feedback improves the trauma resuscitation process [58]. TVR benefits teamwork, communication, and adherence to protocols [59]. Resident physicians also report improved team function and clinical competency when TVR was implemented [60]. More recently, the video review process has been applied to cardiopulmonary resuscitation (CPR). Recent studies have demonstrated the benefit of video recording CPR in the hospital to provide targeted education and feedback to improve clinical outcomes. 

Brooks et al. conducted a pilot study of CPR video review in a large urban academic emergency department (ED). The recorded videos were reviewed to assess for specific quality metrics, then targeted feedback was emailed to each staff member involved. In addition, CPR videos were reviewed during dedicated resident educational time. The authors found a significant decrease in the pulse check time during CPR and the chest compression fraction [4]. Jiang et al. also initiated a video review process of ED CPR cases. The authors provided a weekly video-based review and education to ED staff. This resulted in a decrease in EMS hands-off time and time to first chest compression [61]. Rolston et al. initiated a bi-weekly CPR video review pilot program with ED staff. The authors found that the video review process resulted in a significant increase in return to spontanesous circulation (ROSC) [62]. Finally, Yamane et al. recorded and reviewed OHCA with ED CPR. The authors again reviewed each video for targeted quality metrics and provided email feedback to the team involved in the case. Videos were also reviewed with residents on a bi-montly basis during dedicated education time. The live video review focused on the duration of pulse checks and the use of point-of-care ultrasound (POCUS) during CPR to assist in pulse checks. The authors found that there was a significant decrease in the pulse check time both with and without POCUS after the intervention [63]. 

### 3.5. AED Unmanned Aerial Vehicle Delivery 

Patients have a greater rate of survival the sooner defibrillation is applied to the onset of ventricular fibrillation (VF) [64]. The success of defibrillation is reduced by about 10% per minute following onset of VF. This contributes to the fact that the survival of out-of-hospital VF cardiac arrest is lower than for in-hospital VF cardiac arrest [65]. To improve the time to defibrillation in out-of-hospital cardiac arrest (OHCA), recent studies have explored the feasibility of deploying unmanned aerial vehicles (drones) to deliver automated external defibrillators (AEDs) in the community. Drones have the potential of delivering AEDs to bystanders in a fast, safe, and easily usable manner. Drone delivery can also access remote and rural locations where community AEDs are inaccessible [66]. 

A 2019 study by Sanfridsson et al. simulated the delivery of an AED by drone to bystanders performing CPR on a manikin. The authors found that drone delivery of an AED was safe and feasible, with the fastest times from collapse to defibrillation completed when participants were in pairs versus on their own [67]. To further investigate drone delivery, Bogle et al. designed a mathematical model to optimize the prime locations for 500 drone docking stations in the state of North Carolina. When compared to the standard emergency medical system without drones, the simulated drone system decreased the time to defibrillation from 7.7 min to 2.7 min. The simulated drone system also doubled the expected survival rate [68]. Boutilier et al. supported these findings when the authors modeled a drone delivery system for southern Ontario. In rural areas, drone delivery was 10 min faster than EMS delivery, and in urban areas drone delivery was 6 min faster [69]. Derkenne et al. further demonstrated the utility of drone delivery in a large urban environment. The authors created a model to compare drone delivery versus standard EMS AED delivery in Paris and found that drones delivered AEDs approximately three minutes faster, both during the day and at night [3]. The feasibility of drone use at night was further proved by Scholz et al. The deployed drones delivered AEDs both during the day and at night. They found that there were no significant differences in length of time or safety events between night and day. The authors also found that the drones could be operated automatically (without a remote driver) successfully both during the day and at night [70]. Mathematical modeling can also be applied to targeted geographical locations to identity places for drone docking sites to optimize time to defibrillation [71]. While mathematical modeling and simulations have well established the utility of drones, studies are lacking that demonstrate drones used in real cases. In a novel study, Schierbeck et al. deployed drones to 14 cases of real-life OHCA. The drones arrived prior to EMS 64% of the time and were successfully used 92% of the time [72]. In order for bystanders to successfully use AEDs delivered by drones, Sedig et al. studied public perception of drones. They found that drones are perceived as acceptable delivery models, however, the authors did find that use of an AED in general did cause some anxiety in rural participation in the study. This demonstrates that a successful drone program does require community engagement and education [73]. These simulated, modeled, and early pilot studies demonstrate that drone delivery of AEDs is safe, feasible, and fast. However, further research is needed on real-life suspected OHCA drone AED delivery on a larger scale. 

### 3.6. Point-of-Care Ultrasound

Point-of-care ultrasound (POCUS) use during cardiac arrest in the hospital setting is well studied. POCUS can diagnose reversible causes of cardiac arrest, prognosticate survival, and is associated with improved odds of ROSC [5,74,75,76]. POCUS can also be used to identity reversible causes of cardiac arrest like cardiac tamponade, pulmonary embolism, tension pneumothorax, and hypovolemia [77]. Furthermore, POCUS can identify opportunities for intervention, like pericardiocentesis or thoracostomy, that deviate from the standard ACLS algorithm [5]. However, POCUS is not yet well studied in the prehospital setting. Point-of-care ultrasound (POCUS) use in the prehospital setting has the potential to guide interventions, improve resuscitation, and predict survival in OHCA [78]. 

Emergency medical services (EMS) POCUS use remains rare, but is steadily increasing every year [79]. Fitzgibbon et al. demonstrated that EMS physicians can feasibly obtain and accurately interpret cardiac standstill on POCUS images in the prehospital setting [78]. After a two-hour ultrasound course, physicians who do not have previous experience with ultrasound can use POCUS in the prehospital setting to predict the outcome in cardiac arrest. Cardiac movement on POCUS was significantly associated with survival and cardiac standstill was significantly associated with death at the scene [80]. While these studies demonstrate the feasibility and utility of physicians’ use of prehospital POCUS, Kreiser et al. investigated the use of POCUS by paramedics. The paramedics received a four-hour course on POCUS in cardiac arrest as well as a new protocol for POCUS integrated into the EMS algorithm for OHCA. The authors found that paramedics obtained adequate POCUS images that were interpreted accurately in a significant number of cases. In addition, POCUS also altered patient management and chest compressions were paused for less than 10 s in a significant number of patients [81]. Numerous studies have further demonstrated that paramedics trained in POCUS can obtain adequate ultrasound images and interpret cardiac standstill [82,83,84]. POCUS can also be used in the prehospital setting to improve the location and quality of chest compression in CPR [85]. No studies to date have demonstrated that prehospital POCUS use improves patient outcomes. 

## 4. Discussion

Innovations in medical technology provide novel opportunities to advance and improve cardiopulmonary resuscitation both in the prehospital and hospital setting. The utility, safety, and feasibility of such technologic advancements must be prudently analyzed and the applied clinical outcomes carefully validated in order to inform clinical practice. A comprehensive literature review of innovative technologies in the field of cardiopulmonary resuscitation highlights six critical technologies: extracorporeal membrane oxygenation, real-time feedback devices, smart devices, video review, AED drone delivery, and point-of-care ultrasound (Table 1).

The utility of ECPR has been studied in numerous systematic and meta-analyses, with an emphasis on survival and neurologic outcomes for patients with promising results. While ECPR may improve survival, its widespread use can be challenging given it requires considerable resources, training, equipment, and personnel, which leads to a highly expensive process. Coordinated healthcare delivery systems will need to consider a protocol for when to implement ECPR, as the data suggest that early initiation of ECPR for patients with an initial shockable rhythm leads to higher rates of survival with meaningful neurologic and functional outcomes. 

The use of various forms of technology during CPR has changed the way bystanders, EMS, and other healthcare professionals deliver CPR. With smartphones and watches providing real-time feedback to reinforce proper technique and ensure adequate compression depth and rate are achieved, there is a trend towards improved outcomes for patients who suffer cardiac arrests both in and out of the hospital. New technologies are developed to further improve how we administer CPR and are shaping the way we teach students and professionals. Manikins which have audiovisual feedback capabilities are becoming standard to practice during simulations and training sessions and have shown to help improve acquisition and retention of essential CPR skills. 

CPR video review provides a unique opportunity to analyze quality metrics, provide targeted feedback, and improve patient outcomes. The greatest limitation to video review is the physical camera installation process. Once achieved, expert review of cardiac arrest videos can analyze adherence to American Heart Association quality metrics. Pilot studies have demonstrated that targeted feedback based on video review to the multidisciplinary staff involved in each case can reinforce positive aspects of the team’s performance and provide specific areas for improvement. In-person multidisciplinary video review has also been proven to increase the rate ROSC and targeted CPR metrics. 

One of the greatest barriers to survival in OHCA is the time to defibrillation. AEDs are often inaccessible at the time and location of arrest for bystanders. Drone delivery of AEDs has been proven to safe for bystander use and accepted by the community at large. Mathematical modeling can isolate the ideal number and locations of drone placement to achieved targeted time to defibrillation goals. Pilot studies have also demonstrated that drone delivery as compared to standard bystander retreatment or EMS delivery has superior time to defibrillation both during the day and at night. Drone delivery can decrease valuable seconds and minutes of down time in OHCA. Perhaps the biggest barrier to drone systems is that they would require significant governmental and medical infrastructure development as well and financial investment.

Expert use of POCUS during cardiac arrest is well established in the medical literature to improve rates of ROSC, identify and assist in reversible causes of arrest, and to prognosticate survival. More recent studies have shown that EMS physicians and paramedics novice to POCUS can capture and interpret POCUS images during OHCA. POCUS can also be used to evaluate the quality of chest compressions and can be performed in less than 10 s during pulse checks. Further studies are needed to establish if prehospital POCUS improves patient outcomes. The invention of portable ultrasound machines that are lightweight and durable, with a general trend in decreasing cost, is a valuable opportunity for more EMS systems to invest in prehospital POCUS. 

## 5. Limitations

An inherent limitation to studying novel technologies in CPR is the lack of large randomized control trials with adequate power. While some interventions like ECMO and POCUS in the hospital have been more extensively studied, other technologies like drone AED delivery have not been externally validated by larger studies. The feasibility and safety of new technologies and early pilot studies provide valuable information on specific quality metrics, however, further research is required for many of the technologies reviewed to demonstrate improved patient outcomes and survival. Multiple reviewers screened the included articles, however, author discretion to include articles outside of the initial MeSH search also allows the possibility of inclusion bias. 

## 6. Conclusions

The use of technology to improve CPR quality has been demonstrated with the use of various forms of technology, including: smart devices, real-time feedback devices, drones, ECMO, POCUS, and video review. Smartphones and watches and newer devices aimed at providing feedback to the individual administering chest compressions have a positive effect on compression rate, depth, and allowance of chest recoil. Real-time device feedback instruments have improved technique immediately while CPR is ongoing and have improved retention of skills with proper technique for both adult and infant CPR. The incorporation of video review has further solidified the need for frequent reassessment and reinforcement of CPR skills and has promising results for improvement in patient outcomes. Drones are being actively studied as delivery methods for AEDs with improved time to defibrillation and therefore greater survival in OHCA. POCUS and ECMO are highly studied in the field of CPR with overall positive data demonstrating improved outcomes for patients when used early in the cardiac arrest and can help guide resuscitation and intervention. More research is needed into the implementation of ECMO as a standardized practice during CPR and on POCUS use for EMS providers. 

## Figures and Tables

**Table 1:** Summary of strengths and weaknesses of various forms of technology utilized during CPR.

	Strengths	Weaknesses
Extracorporeal Membrane Oxygenation (ECMO)	Improved patient survival and neurologic outcomes compared to standard ACLS	Patient-specific factors affect success of intervention Expensive, requires staff training and resources to implement
Point-of-Care Ultrasound (POCUS)	Diagnose reversible causes of arrest and guide intervention Improve ROSC Prognosticate survival Lightweight and portable	Not well studied in prehospital setting
Video Review	Improved teamwork and clinical outcomes Decrease in pulse check time, increase in ROSC	Video review takes time and feedback is delayed Requires physical camera installation
Drone	Can deliver AEDs to bystanders and access remote and rural locations Decreased time to initiate shock for OHCA Faster arrival of AED on scene compared to EMS	Pilot and simulated data available, more real-life studies are needed Public perception of drones affects willingness to accept as a delivery system for AEDs Requires government and medical infrastructure and financial investment
Real-time Feedback Devices		
AEDs	Visual feedback provided which leads to improvement in depth, rate, and chest recoil during CPR	Financial concern
Portables (Cardio First, CPRmeter, CPR assist)	Improvement in chest compression depth and rate Decreased perceived workload for team leaders during CPR when a device is used	Inconsistent across devices Requires proper placement of device to provide accurate feedback Can cause delay in initiation of CPR
Manikins	Reduction in hands-off time, and improvement in all CPR metrics Improved retention of CPR skills Less biased feedback compared to instructor training	Differences between adult and infant manikins and type of feedback given (audio, visual, etc)
Smart Devices		
Phones	Apps can improve bystander CPR technique (rate and depth of compressions) Alerts civilians to nearest AED and location of cardiac arrest for faster on scene arrival than EMS and quicker shock administration	Can be difficult to hold phone during CPR Requires app to be downloaded
Watches	Uses vibration function to provide feedback on chest compression rate and depth Users feel more confident administering CPR	Financial concern
Glasses	Improvement in wearer performance of basic life support (BLS) and use of AED to administer shock	No improvement in CPR quality
Rings	Improvement in chest compression depth Useful as a feedback device for infant CPR	Pilot data, require more studies

## Data Availability

The data presented in this study are openly available in PubMed and Scopus.
